# Intra-osseous migration in calcific rotator cuff tendinopathy- a novel depiction of temporal evolution on multimodality imaging

**DOI:** 10.1259/bjrcr.20210156

**Published:** 2022-01-20

**Authors:** Samir Mustaffa Paruthikunnan, Mathieu Boily, Marie-Hélène Martin, Adel Assaf, Rehana Jaffer

**Affiliations:** 1 York and Scarborough Teaching Hospitals NHS Foundation Trust, York, United Kingdom; 2 Department of Radiology, McGill University Health Centre, McGill University, Montreal, Quebec, Canada

## Abstract

We present a case of calcific tendinopathy of the rotator cuff with intraosseous migration of the calcification, treated with ultrasound-guided bursal steroid injection and followed up with multiple imaging modalities for a year following the initial presentation. The radiographs, ultrasound, CT, nuclear scintigraphy, and MRI images demonstrate the temporal evolution of the intraosseous migrated calcium and show how this pathology, in its acute phase, can mimic other pathologies like osteoid osteoma. The follow-up imaging also illustrates how the migrated intraosseous focus of calcification took a much longer time to heal compared to its intratendinous counterpart, possibly leading to the protracted course of recovery. This report also highlights a previously undescribed pattern of healing of the intraosseous migrated calcium on multiple imaging modalities.

## Introduction

Calcific tendinopathy is a relatively common condition related to the deposition of calcium hydroxyapatite crystals within the tendons.^1^ This condition often involves the rotator cuff tendons, especially the supraspinatus tendon, in middle-aged females. Although benign and self-limiting, it can be an excruciatingly painful condition by inciting inflammatory changes. Intraosseous migration of these tendinous calcifications is relatively uncommon but well-documented in the literature.^1^

Although this pathology is evident on radiographs, CT and ultrasonography, MRI is often requested by clinicians in the setting of non-traumatic shoulder pain. The intraosseous manifestations of migration of intratendinous calcification may mimic a bone tumour or infection, especially on MRI. At times, further imaging like bone scintigraphy may also be requested in such a situation, further adding to the diagnostic dilemma. The challenge in making the diagnosis on MRI is exacerbated by the knowledge gap in the temporal evolution of these lesions on various modalities. To our knowledge, there is only one article in the literature describing the evolution of these migrated intraosseous calcium deposits on radiography 1 year after the presentation, with no articles describing the associated bone changes over time on MRI.^2^ Our case report attempts to bridge this knowledge gap and present the temporal evolution of intraosseous migration of rotator cuff tendinous calcifications on multiple modalities 1 year after initial presentation and following treatment by ultrasound-guided bursal injection of steroid.

## Clinical presentation

A 36-year-old, right-hand-dominant lady presented to her general physician with acute onset severe pain in her left shoulder. There was no history of trauma or surgery to the shoulder. She did not respond to a trial of physiotherapy and was referred to an orthopaedic surgeon by her general physician. Physical examination by the surgeon revealed mild weakness in internal rotation of the left shoulder with generalised restriction in shoulder movements due to pain. Her blood counts and serum inflammatory markers were within normal limits.

### Imaging findings, follow-up and treatment

**Figure 1.**
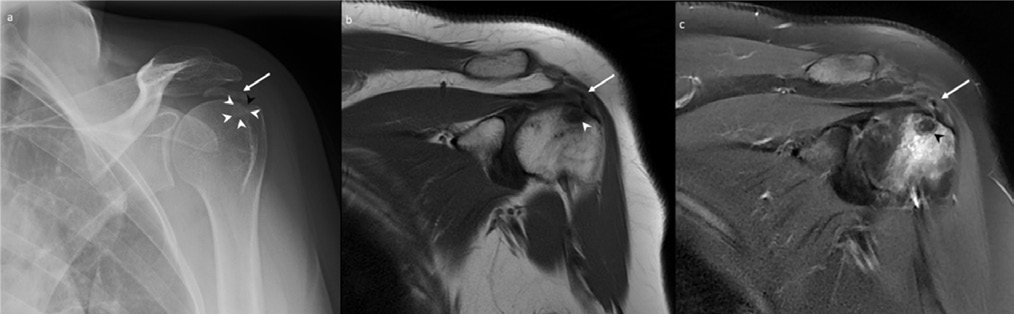
A 36-year-old female with calcific tendinopathy of the left rotator cuff and intraosseous migration of the calcium. Figure 1a is the frontal radiograph depicting a 2.1 cm amorphous calcification within the left supraspinatus tendon, close to the adjacent greater tuberosity (white arrow). In addition, faint sclerosis (white arrowheads) with a subtle cortical erosion (black arrowhead) is also seen at the greater tuberosity (white arrowhead). Figure 1b and c are the coronal *T*_1_ weighted and coronal *T*_2_ fat-saturated MR images, respectively, demonstrating a partially imaged calcific tendinopathy of the supraspinatus tendon (white arrows) with a focus of low signal (white arrowhead in Figure 1b and black arrowhead in Figure 1c) within the adjacent greater tuberosity and significant surrounding bone marrow oedema, suggesting intraosseous migration of the tendon calcification.

Radiographs of the left shoulder confirmed a 2.1 cm amorphous calcification within the supraspinatus tendon. The calcification was in close relation to the adjacent greater tuberosity. Faint sclerosis with a subtle erosion with sclerosis was seen at the greater tuberosity on the radiographs (Figure 1a). There was no fracture, periosteal reaction or joint space narrowing seen. The corresponding MRI (performed on a 1.5 T General Electric MRI scanner) again demonstrated the calcific supraspinatus tendinopathy, with associated mild oedema surrounding the calcium deposit. A focus of low T1 and T2 signal intensity within the greater tuberosity adjacent to the tendon calcification, corresponding to the erosion on the radiographs. In addition, there was significant perilesional bone marrow oedema within the greater tuberosity (Figure 1b and c). This was considered to be a focus of intraosseous migration of the tendon calcification. There was no evidence of a tendon tear, joint effusion, or any soft tissue mass or collection to support alternate diagnoses.

**Figure 2.**
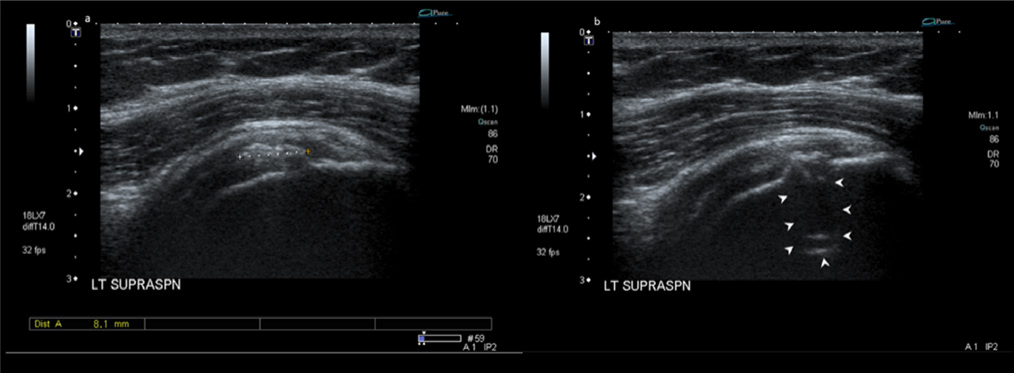
A 36-year-old female with calcific tendinopathy of the left rotator cuff and intraosseous migration of the calcium. Figure 2a and b are follow-up ultrasound images of the left supraspinatus tendon (2 months after the initial presentation) which show a significant reduction in the size of the tendon calcification (which measures 8 mm) with a focal cortical erosion within the greater tuberosity adjacent to the calcification (white arrowheads).

The patient was referred for a percutaneous ultrasound-guided lavage of the tendon calcification 2 months after the MRI. The ultrasound evaluation showed a significant reduction in the size of the tendinous calcification, which measured only 8 mm in the largest dimension and was not amenable for a lavage (Figure 2a). The ultrasound also demonstrated a focal cortical erosion within the greater tuberosity adjacent to the calcification (Figure 2b). The patient received an ultrasound-guided injection of 40 mg methylprednisolone mixed with 1 cc of 1% lidocaine and 1 cc of 0.5% bupivacaine into her subacromial–subdeltoid bursa in the same sitting.

**Figure 3.**
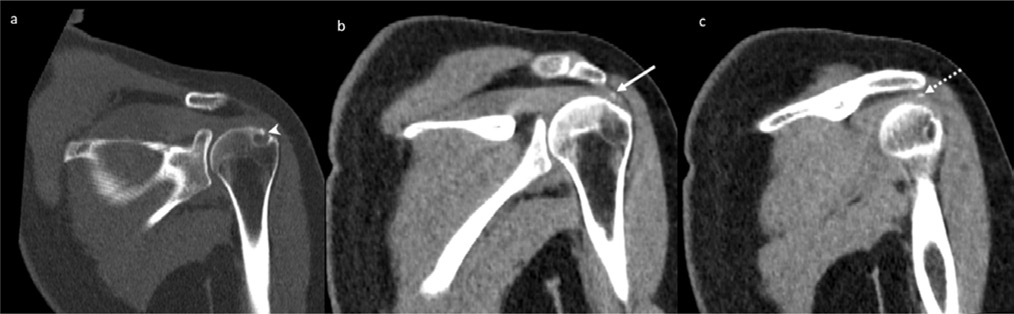
A 36-year-old female with calcific tendinopathy of the left rotator cuff and intraosseous migration of the calcium. Figure 3a, b and c are serial coronal CT images performed 5 months after the ultrasound scan and the ultrasound-guided injection. Figure 3a shows a 1 cm osteolytic focus in the left greater tuberosity (white arrowhead), corresponding to the sclerotic area in the radiograph from Figure 1 and erosion on the ultrasound from Figure 2. Figure 3b shows further reduction in the size of the calcific tendinopathy of the supraspinatus (white arrow), measured around 6 mm at the time of the CT scan. Figure 3c shows faint calcifications in the subacromial-subdeltoid bursa (dotted white arrow), suggesting a co-existent bursal migration of calcification.

**Figure 4.**
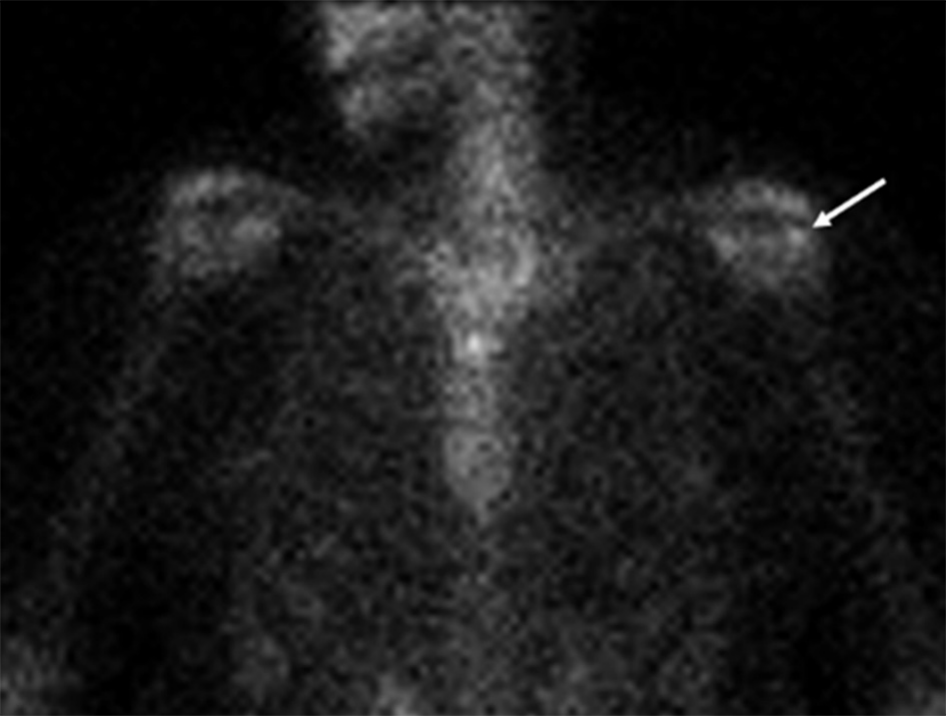
A 36-year-old female with calcific tendinopathy of the left rotator cuff and intraosseous migration of the calcium. Figure 4 is the coronal AP projection of the ^99m^Tc bone scan performed 8 months after the initial presentation, demonstrating a focal mildly increased bone tracer activity at the site of the osteolytic lesion in the left greater tuberosity (white arrow), suggesting a mildly active bone process.

The patient only had a mild reduction in her symptoms following this treatment. In addition, she had significant persistent pain limiting her activities, which got worse at night. 5 months later (*i.e.* 7 months after the first MRI scan), the patient returned to her orthopaedic surgeon, who requested a CT scan of the shoulder. The CT demonstrated a 1 cm osteolytic focus in the region corresponding to the previously demonstrated T1 and T2 low signal focus on MRI and erosion on radiograph and ultrasound (Figure 3a). The tendon calcification had further reduced in size as well as density. The largest dimension of the calcium deposit measured around 6 mm at the time of the CT scan (Figure 3b). Faint calcifications were also seen in the subacromial–subdeltoid bursa, suggesting a co-existent bursal migration of calcification (Figure 3c). Due to the clinical presentation of nocturnal pain and the CT features of an osteolytic lesion without any direct visible osseous extrusion of the tendon calcification, the possibility of an osteoid osteoma was also raised at this point. Accordingly, the orthopaedic surgeon requested a ^99m^Tc nuclear bone scan, which was performed 6 weeks later, and showed focal mildly increased bone tracer activity at the site of the osteolytic lesion (Figure 4). However, the uptake was significantly lower than expected for an osteoid osteoma and was considered to represent a mildly active process within the greater tuberosity.

**Figure 5.**
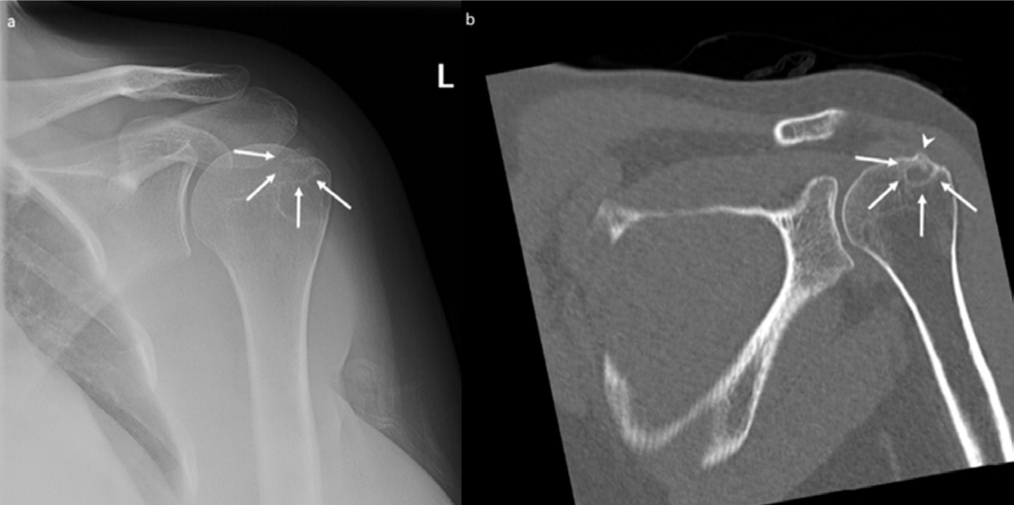
A 36-year-old female with calcific tendinopathy of the left rotator cuff and intraosseous migration of the calcium. Figure 5a and b are the follow-up coronal radiographs and coronal CT section of the left shoulder, performed almost a year after the initial presentation, showing the osteolytic focus with interval ossification around it (white arrows). Figure 5b also shows interval healing of the cortical erosion with some bony remodelling at the site of the previous erosion (white arrowhead). The tendon calcification is no longer identified.

**Figure 6.**
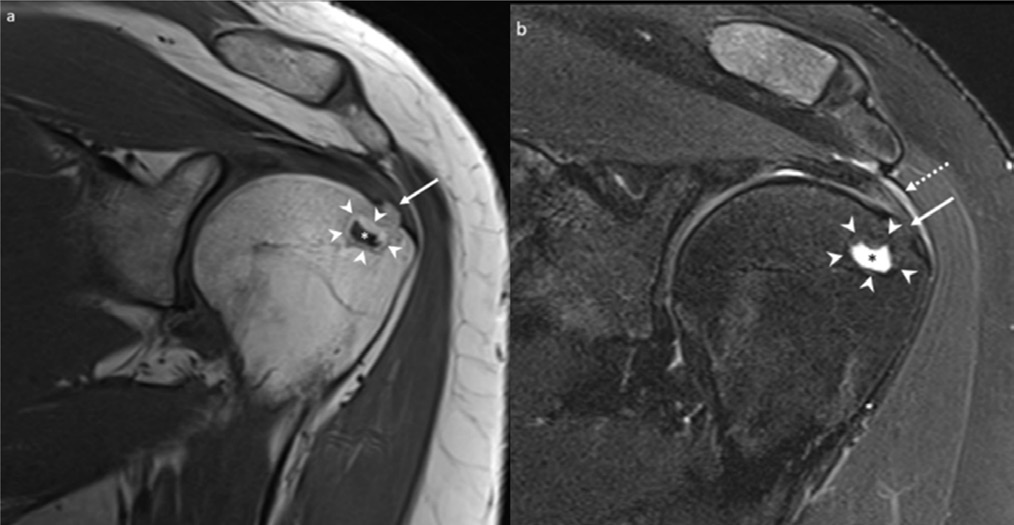
A 36-year-old female with calcific tendinopathy of the left rotator cuff and intraosseous migration of the calcium. Figure 6a and b are coronal *T*_1_ weighted and coronal *T*_2_ fat-saturated MR images showing the formation of a peripheral fat signal rim (white arrowheads in Figure 6a and b) with central fluid signal (white asterisk in Figure 6a and black asterisk in Figure 6b) at the site of the previously demonstrated low T1 and T2 signal within the greater tuberosity of the left humerus, consistent with interval healing of the intraosseous focus of migrated calcium. The overlying cortical bone remodelling is also evident in the MR images (solid white arrows). There is also interval resolution of the surrounding bone marrow oedema, depicted in Figure 1b and c. Figure 6b also shows residual mild supraspinatus tendinosis with thickening of the subacromial–subdeltoid bursa (dotted white arrow).

Since the CT and bone scintigraphic findings were not typical of an osteoid osteoma, the patient was started on conservative management and follow-up radiographs and a second CT scan were performed 5 months after the bone scan and almost a year after the initial presentation and the first MRI scan. The radiograph and CT demonstrated the same osteolytic focus within the greater tuberosity of the proximal humerus, which was stable in size but showed interval ossification along its periphery (Figure 5). The previously demonstrated cortical erosion had also healed in the follow-up CT with evidence of bony remodelling of the cortex. The tendon and bursal calcifications identified in the previous CT were not seen in this CT scan. The patient also underwent an MRI a month later, which showed the development of a peripheral rim of fatty signal with central high T2 signal in the region corresponding to the low T1 and T2 signal area in the previous scan, with surrounding cortical bone remodelling, in keeping with healing (Figure 6). The bone marrow oedema had resolved in the follow-up MRI.

In summary, the follow-up imaging features ruled out the possibility of a neoplastic bone lesion. The lesion was consistent with a migrated intraosseous calcific tendinopathy, demonstrating features of healing. The patient’s symptoms had also improved with conservative therapy, paralleling the imaging findings and suggesting her persistent symptoms post-percutaneous bursal steroid injection were related to the bone and bursal involvement of the calcific tendinitis.

## Discussion

Accounting for almost 7% of all painful shoulder conditions, the presentation of calcific tendinopathy of the rotator cuff may vary from asymptomatic to severe pain. In addition, the symptomatology in calcific tendinopathy does not always correlate with the imaging findings.

Uhthoff et al^3^ proposed that calcific tendinitis evolved through three stages: pre-calcific, calcific, and post-calcific. In the pre-calcific stage, the tendon, especially the relatively hypovascular “critical zone”, sustains a hypoxic and mechanical injury resulting in fibrocartilaginous metaplasia. During this stage, the patients are essentially asymptomatic or complain of mild pain and reduced range of motion. The second stage, *i.e*. the calcific stage, is further divided into formative and resorptive phases. The formative phase is the phase of actual calcium deposition mediated by the chondrocytes, while in the resorptive phase, there is vascular weaving in the affected area followed by macrophages attempting phagocytosis of the calcium deposits. This phase is characterised by increased intratendinous pressure, which leads to acute pain and disability, especially if the calcium leaks into the subacromial–subdeltoid bursa. Finally, the post-calcific stage is characterised by tendon tissue remodelling by fibroblasts forming scar tissue. The pain persists during this phase, although less severe than the resorptive phase. These phases vary in duration, especially the latent period between the formative and resorptive phases.

During the resorptive phase, the intratendinous calcification can migrate into the surrounding structures such as bone, subacromial–subdeltoid bursa or even into the adjacent muscles. The exact mechanism of intraosseous migration is not well understood. However, it is postulated that enzymatic action causes lysis of cortical bone of the greater tuberosity, through which calcium salts can penetrate the bone and produce an inflammatory reaction of the adjacent bone marrow.^4^ A histopathological evaluation performed in a few of these lesions confirmed the intense inflammatory reaction incited by the calcified material in the bone marrow, with one of the authors mentioning histiocytic inflammation with giant cells and decalcified areas.^1^

The radiographic features of intraosseous calcium are often subtle and may appear as cortical erosions or irregularities, while rarely the migrated calcium may appear as a dense or sclerotic focus in the proximal humerus.^5^ However, the radiographs are frequently poor predictors of intraosseous migration. In our case, the radiograph did show subtle sclerosis at the site of intraosseous migration initially, which became lytic subsequently on follow-up.

CT detects the signs of intraosseous migration, such as cortical erosions, with much more accuracy. There are varying reports from the literature suggesting that intraosseous calcium may result in an osteosclerotic or an osteolytic lesion within the humeral tuberosity.^4,6,7^ In our case, the lesion was osteolytic. We believe that the lesion appeared osteosclerotic in the early phase, with the enzymatic processes leading to resorption of the calcification and eventually resulting in a lucent lesion at the site of intraosseous migration. Our patient’s follow-up CT images show that the lesion shows healing by reossification along the periphery. Although healing by ossification is a known phenomenon in treated bone lesions,^8^ this pattern of spontaneous healing of intraosseous calcific tendinopathy has not been previously described in the literature to our knowledge.

Ultrasonography is very useful in detecting calcific tendinopathy, appearances of which are well-documented in literature and beyond the scope of this report. Ultrasound appearance of intraosseous migration is intrinsically suboptimal due to lack of bone penetration. However, ultrasound can detect cortical erosions with/without subcortical intraosseous lesions with reasonable success. Establishing continuity between the tendinous calcification and the intraosseous component is essential for establishing a diagnosis. Doppler evaluation may also show hyperemia around the calcification and, in acute stages, around the erosion.^9^

When performed due to clinical suspicion of an aggressive bony lesion, bone scintigraphy may show focal increased uptake at the site of intraosseous migration.

Although MRI is considered less sensitive than CT or radiographs to detect calcifications, it is highly sensitive in detecting the reactive inflammatory changes in the soft tissues and the bone due to the calcifications.^1^ The specific MRI features of intraosseous migration of tendinous calcifications are:

1. Erosion of the greater tuberosity associated with an intraosseous hypointense focus in multiple sequences, which corresponds to the migrated intraosseous calcium.
2. Inflammatory oedema in the perilesional bone marrow and adjacent soft tissue.^4^

In our case, the intraosseous focus returned a typical low signal in the initial MRI during the acute stage, when the migrated calcium was dense enough to appear sclerotic on the radiographs. However, on the subsequent MRI scan, the migrated deposit showed a higher signal on T2, suggesting interval resorption of the migrated calcium. The lesion also demonstrated a peripheral rim of fatty signal with overlying bone remodelling, a sign of healing, along with a complete resolution of the bone marrow oedema, which virtually excluded any aggressive bone lesion.

Our report shows that a CT scan accurately depicts the intraosseous migration of tendon calcification. Nevertheless, CT features can be misleading, especially once the calcification has been resorbed and the cortical erosion begins to heal. The MRI appearance is also highly variable, depending on when the scan is performed. However, MRI is less sensitive than CT for detecting tendon or intraosseous calcifications.

Our patient was treated with percutaneous ultrasound-guided bursal steroid injection since her tendinous calcifications had significantly reduced in size to be amenable for a lavage, partly due to ongoing resorption and partly due to the continued intraosseous and possibly also bursal migration. Despite the injection, she only had a mild reduction in her symptoms. Unsatisfactory results following the percutaneous treatment made the clinician perform additional imaging and raise the suspicion of a possible underlying bone lesion, since there was an improvement in the size and density of the tendinous calcifications while her symptoms persisted. A review of recent literature reveals that the presence of intraosseous migration of calcific rotator cuff tendinopathy is associated with a less satisfactory outcome with percutaneous treatment than an uncomplicated rotator cuff calcific tendinopathy.^10^

In conclusion, this case report highlights a previously undescribed evolution in the appearance of intraosseous migrated calcium over time, with the calcium deposit initially appearing dense in the acute phase on radiographs, with corresponding low signal on MRI. Over the next few months, the focus became osteolytic on CT and showed a higher T2 signal on MR. The healing process began from the periphery in the form of a rim of fat signal, surrounding ossification and bony remodelling. In the acute stage, extensive perilesional bone marrow oedema was also identified on MRI, which later resolved. Despite percutaneous treatment for managing the pain due to the calcific tendinopathy, the intraosseous migration took significantly longer to heal. In concordance with the imaging appearance, the patient also had a less satisfactory result following percutaneous intervention for pain management. Awareness of the temporal evolution of intraosseous migration on imaging is essential to make the correct diagnosis, avoid unnecessary aggressive management, and prognosticate the response to percutaneous treatment of calcific tendinopathy.

## Learning points

1. Intraosseous migration of calcific tendinopathy can have a highly variable appearance on imaging based on the duration since the onset of the pathology.
2. The healing process of the migrated intraosseous calcific focus starts along the periphery of the focus, in the form of fatty marrow proliferation followed by ossification.
3. Patients having calcific tendinopathy with intraosseous migration can have a more protracted course of improvement, even with percutaneous interventions.
4. CT most accurately detects intraosseous migration, especially in the early stages. However, the appearances can be confusing once healing sets in, with the onset of calcium resorption.
5. MRI features of intraosseous migration vary depending on when the scan is performed during the course of the disease. MRI is not as sensitive as CT to detect tendon or intraosseous calcifications.
